# Interleukin-22 Exerts Detrimental Effects on Salivary Gland Integrity and Function

**DOI:** 10.3390/ijms232112997

**Published:** 2022-10-27

**Authors:** Jing Zhou, Shoko Onodera, Yang Hu, Qing Yu

**Affiliations:** 1 The Forsyth Institute, 245 First Street, Cambridge, MA 02142, USA; 2 Department of Biochemistry, Tokyo Dental College, 2-9-18 Kanda Misaki-chou, Chiyoda-ku, Tokyo 101-0061, Japan; 3 Department of Oral Medicine, Infection and Immunity, Harvard School of Dental Medicine, 188 Longwood Avenue, Boston, MA 02115, USA

**Keywords:** Sjögren’s syndrome, sialadenitis, autoimmune exocrinopathy, non-obese diabetic mice, salivary gland epithelial cells, cytokine

## Abstract

Interleukin-22 (IL-22) affects epithelial tissue function and integrity in a context-dependent manner. IL-22 levels are elevated in salivary glands of Sjögren’s syndrome (SS) patients, but its role in the pathogenesis of this disease remains unclear. The objective of this study is to elucidate the impact of IL-22 on salivary gland tissue integrity and function in murine models. We showed that IL-22 levels in sera and salivary glands increased progressively in female non-obese diabetic (NOD) mice, accompanying the development of SS. Administration of IL-22 to the submandibular glands of NOD mice prior to the disease onset reduced salivary secretion and induced caspase-3 activation in salivary gland tissues, which were accompanied by alterations in multiple genes controlling tissue integrity and inflammation. Similarly, IL-22 administration to submandibular glands of C57BL/6 mice also induced hyposalivation and caspase-3 activation, whereas blockade of endogenous IL-22 in C57BL/6 mice treated with anti-CD3 antibody mitigated hyposalivation and caspase-3 activation. Finally, IL-22 treatment reduced the number of viable C57BL/6 mouse submandibular gland epithelial cells cultured in vitro, indicating a direct impact of this cytokine on these cells. We conclude that IL-22 exerts a detrimental impact on salivary gland tissues.

## 1. Introduction

Normal saliva and tear production are critical for maintaining oral and eye health. In Sjögren’s syndrome (SS), a chronic autoimmune condition that affects 2–4 million Americans, the integrity and secretory function of salivary and lacrimal glands are impaired, leading to dry mouth, dry eyes and various systemic health problems [1,2,3]. Cytokines produced by aberrantly functioning immune and non-immune tissue cells in the SS setting, which are elevated in both human patients and mouse models, play crucial roles in promoting or regulating the development of SS pathologies [1,2,3]. Some of these cytokines have the ability to directly act on exocrine gland epithelial tissues and exert detrimental or protective effects [4,5,6,7,8,9,10]. Studies by our group and others have shown that TNF-α and IFN-γ can directly cause salivary gland epithelial tissue apoptosis, tight junction defects and secretory dysfunction [11,12,13,14,15]. Nevertheless, the role of other epithelial-affecting cytokines in this autoimmune exocrinopathy is poorly understood and awaits investigation [4,8,16].

IL-22 is an IL-10 family cytokine produced by various immune cells, including Th17 and Th22 cells, NK cells and innate lymphoid cells, as well as certain non-immune tissue cells, in a context-specific manner [17,18,19,20]. IL-22 mostly targets non-immune cells such as epithelial cells and keratinocytes of barrier tissues, and liver and pancreatic cells [17,18,19,20]. Binding of IL-22 to its receptor, which consists of IL-22R1 and IL-10R2 subunits, leads to activation of JAK-STAT3 and p38 MAPK signaling pathway to facilitate cell survival and tissue repair. IL-22 has widely reported anti-microbial, tissue-protective and anti-inflammatory effects on its target tissues in various inflammatory disease conditions, including inflammatory bowel disease, airway inflammation, pancreatitis and hepatitis [17,18,19,20]. However, the signaling and functional properties of IL-22 are highly complex and context-dependent. In psoriasis, IL-22 exacerbates tissue-pathologies and inflammations [17,19]. In *T. gondii*-induced intestinal inflammation, IL-22 enhances IL-18 production by epithelial cells to promote the T helper 1 (Th1) response and the consequent IFN-γ production [21]. Moreover, IL-22 signaling properties and downstream actions can be altered, sometimes dramatically, by the surrounding cytokines in the local environment. Priming with type 1 IFNs significantly enhances the proinflammatory and tissue-detrimental properties of IL-22 by augmenting STAT1 while attenuating STAT3 activation, and in an experimental lung inflammation and injury model, IL-17 converts IL-22 activity from tissue-protective to proinflammatory in nature [22,23,24]. Importantly, a recent study reveals that IL-22 plays a detrimental role in chronic colitis through enhancing the endoplasmic reticulum stress response in colonic epithelial cells [25], adding a new mechanism for the context-dependent pathogenic actions of IL-22 in epithelial tissues. 

Emerging evidence has begun to uncover a functional importance of IL-22 in exocrine gland inflammatory diseases. In patients with non-SS type dry eye diseases, IL-22 levels are increased in lacrimal fluids and negatively correlated with disease severity [5]. IL-22 attenuates IL-17-mediated ocular tissue inflammation and pathologies in a desiccation-induced mouse model of dry eye disease [26]. In SS patients, both IL-22 and IL-22R1 are present at elevated levels in salivary glands, with immune cells being the major IL-22 producers and IL-22R1 detected on mononuclear immune cells, epithelial cells and endothelial cells [4,8,10]. Serum IL-22 concentration is also markedly higher in SS patients compared to the control subjects and positively correlates with SS severities [4,8,10]. Moreover, in a viral infection-induced mouse model of SS-like sialadenitis, IL-22 deficiency leads to impaired B cell accumulation and tertiary lymphoid organ (TLO) formation within salivary glands and reduced autoantibody production, with the effect associated with defective upregulation of chemokines CXCL12 and-13 in salivary gland tissue cells in the TLOs [27]. Hence, a complex role of IL-22 in exocrine gland disorders and SS disease is emerging, with both protective and detrimental effects reported, and demands a better characterization of its actions in these contexts. 

This study is undertaken to determine the impact of excessive IL-22 on salivary gland integrity and cellular and secretory function under both the non-inflammatory, steady-state condition in C57BL/6 mice, and the SS-prone condition in the non-obese diabetic (NOD) murine model. The Balb/c mice were also utilized to serve as approximate controls for the NOD mice. Our results indicate that IL-22 exerts a detrimental and pathogenic effect on salivary gland integrity and function under both non-inflammatory, steady-state condition and the SS inflammatory condition. 

## 2. Results

### 2.1. Elevation of IL-22 in Salivary Glands of NOD Mice Promotes Tissue Apoptosis and Secretory Dysfunction

**Figure 1:**
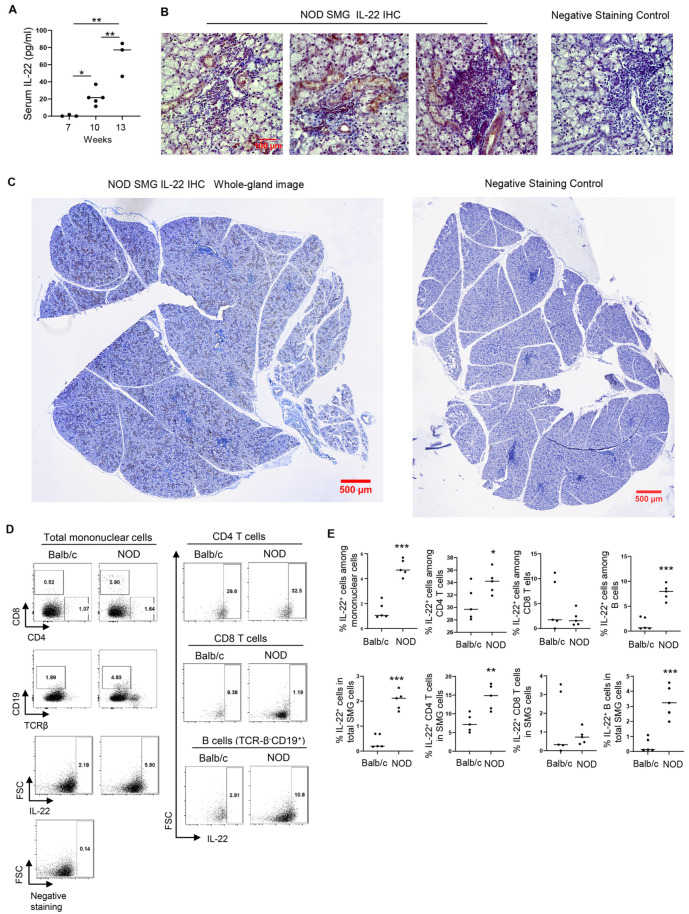
IL-22 expression in the NOD mice. (**A**) ELISA measurement of serum IL-22 concentrations in female NOD mice at various ages (n = 6–12 per group). Each black dot represents the measurement from an individual mouse. (**B**) Representative images of immunohistochemical staining of IL-22 in the submandibular glands (SMGs) of 13-week-old NOD female mice (n = 4; Magnification: 400×). (**C**) Whole-gland images of the stained samples described in (**B**). (**D**) Dot plots of flow cytometry analysis of lymphocyte populations in SMGs of 13-week-old female NOD mice described in (**B**) above, and in gender- and age-matched Balb/c mice, and IL-22 expression in each population (n = 5 per group). (**E**) Graphs showing the percentages of IL-22-producing cells in different cell populations from the flow cytometric analysis in (**C**) above. Each black dot represents the measurement from an individual mouse. Error bars represent the standard error of mean (SEM). The statistical significance was determined by One-way ANOVA in (**A**) and by two-tailed Student’s *t*-test in (**E**). * *p* < 0.05; ** *p* < 0.01; *** *p* < 0.001.

IL-22 and its receptors are present at elevated levels in salivary glands of SS patients compared to the healthy subjects [4,8], but the significance of such increase in the pathogenesis of SS-like salivary gland inflammation and dysfunction has not been defined. We therefore examined IL-22 expression in female NOD mice that spontaneously develop SS-like salivary gland pathologies and have the initial disease onset around 10–11 weeks of age as we have previously delineated [15,28,29]. ELISA assay showed that serum IL-22 concentrations in female NOD mice increased continuously with age, accompanying the development, onset and persistence of SS (Figure 1A). In agreement with the ELISA results, the IL-22 protein was detected at significant amounts by immunohistochemical staining in SMG tissues of 13-week-old NOD mice that had newly established SS disease, especially in the immune cells in the leukocyte foci and the ductal epithelial cells (Figure 1B,C). Some acinar cells were also stained positive for IL-22, albeit at a much lower frequency compared to the immune and the ductal cells (Figure 1B,C). Flow cytometric analysis of IL-22 production by lymphocyte sub-populations revealed that the percentage of IL-22^+^ cells among SMG mononuclear cells and that among total SMG cells in NOD mice were higher than those in the Balb/c controls, at age 16 weeks (Figure 1D,E). In comparison, the proportion of IL-22^+^ CD4 T cells among SMG mononuclear cells was not altered, and that among total SMG cells was only marginally elevated in NOD mice compared to the Balb/c mice (Figure 1D,E). Interestingly, a significant fraction of B cells (defined as CD19^+^TCRβ^−^) was IL-22^+^, and the percentage of IL-22^+^ B cells among mononuclear cells and that among total SMG cells showed a substantial increase in NOD mice compared to the Balb/c mice, to a greater degree than those of IL-22^+^ CD4 T cells (Figure 1D,E). Hence, IL-22 levels and IL-22^+^ cells increased in female NOD mice with time and exhibited a significant difference compared to the Balb/c mice.

**Figure 2:**
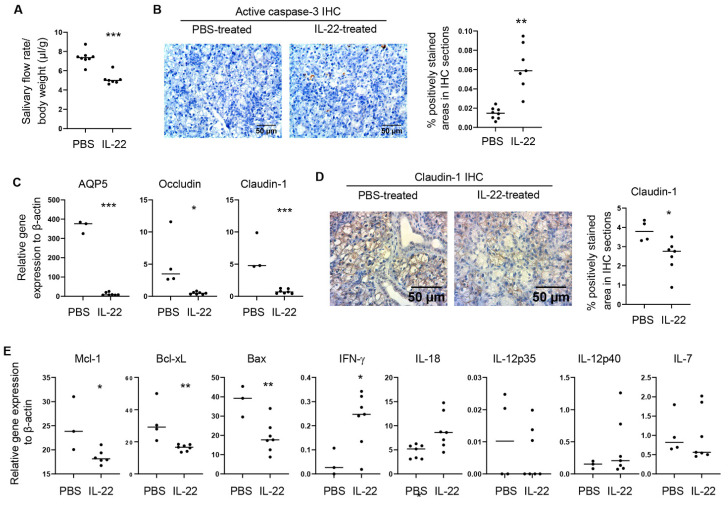
IL-22 administration to SMGs of the NOD mice rapidly induces hyposalivation and tissue apoptosis. Recombinant IL-22 or control PBS was injected into the SMGs of female NOD mice aged 8–9 weeks on day 0 and day 2. The mice were analyzed on day 3. (**A**) Stimulated salivary flow rate normalized to body weight (n = 4–7 per group). (**B**) Left panels, Immunohistochemical staining of active caspase-3 in the SMGs; right panel, the graph shows the mean percentage of positively stained areas in the stained sections. Magnification: 400×. (n = 4–7 per group). (**C**) Real-time qPCR analysis of gene expression in the SMG tissues, presented relative to β-actin. (**D**) Immunohistochemical staining of claudin-1 in the SMGs; right panel, quantification of the percentage of positively stained areas. (**E**) Gene expression in the SMG tissues, presented relative to β-actin. In all the dot plot graphs in this figure, each black dot represents the measurement from an individual mouse. All data are representative or the averages of analyses of 4–7 mice per group. * *p* < 0.05; ** *p* < 0.01; *** *p* < 0.001.

To elucidate the in vivo impact of elevated IL-22 on salivary gland integrity and function in the context of SS, we injected recombinant murine IL-22 directly into the SMGs of female NOD mice aged eight weeks, prior to the initial disease onset. IL-22 was injected to the SMGs twice, two days apart, and the mice were analyzed one day after the 2nd injection. The dosage and injection frequency were selected based on multiple previous reports that demonstrate in vivo efficacy of this regimen in mice [30,31]. Mice that were injected with PBS containing the appropriate low concentration of LPS (to control for the possible endotoxin contamination of IL-22) served as the controls. Compared to the control group, IL-22 treatment led to a marked reduction in stimulated salivary flow rate (Figure 2A) and an increase in the amount of activated caspase-3 in SMG tissues as shown by immunohistochemical staining, indicating augmented tissue apoptosis (Figure 2B). Real-time qPCR analysis revealed that IL-22 also substantially reduced the mRNA amounts of occludin, claudin-1 and aquaporin-5 (AQP5) (Figure 2C), key players in maintaining cell survival, tight junction integrity and normal salivary secretion [12,13,32,33,34,35]. The IL-22-induced diminishment in claudin-1 protein levels was further confirmed by immunohistochemical analysis (Figure 2D). In addition, IL-22 treatment downregulated the expression of anti-apoptotic molecules Mcl-1 and Bcl-xL in SMG tissues, and significantly increased the expression of IFNγ, a critical SS-promoting factor as we and others previously reported [11,28,36,37] (Figure 2E). IL-22-induction of IFNγ was associated with a marked upregulation of IL-18, a cytokine that enhances IFNγ production by Th1 and other immune cells, but not an alteration in the expression of IL-7 or IL-12 (Figure 2E), two other Th1-promoting cytokines. Hence, exogenous IL-22 treatment promotes salivary gland tissue damage and dysfunction and IL-18 and IFNγ expression in the NOD model of SS.

### 2.2. Administration of Exogenous IL-22 Induces Salivary Gland Tissue Apoptosis and Secretory Dysfunction in Normal C57BL/6 Mice

**Figure 3:**
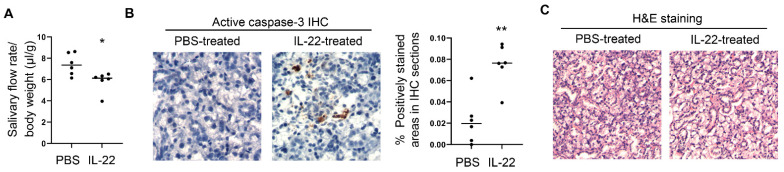
IL-22 administration to SMGs of normal C57BL/6 mice rapidly induces hyposalivation and tissue apoptosis. Recombinant IL-22 or control PBS was injected into the SMGs of female C57BL/6 mice aged 7–12 weeks on day 0 and day 2. Mice were analyzed on day 3. (**A**) Stimulated salivary flow rate normalized to body weight. (**B**) Immunohistochemical staining of active caspase-3 in the SMGs. Magnification: 400×. Graph shows the percentage of positively stained areas. (**C**) H&E staining of SMG sections. In all the dot plot graphs in this figure, each black dot represents the measurement from an individual mouse. All data are the average or representative of 6 samples per group. * *p* < 0.05; ** *p* < 0.01.

We next assessed if exogenously provided IL-22 can also exert a detrimental effect on otherwise normal salivary gland tissues by using normal C57BL/6 mice. Recombinant murine IL-22 was directly injected into the SMGs of female C57BL/6 mice twice, two days apart, and the mice were analyzed 1 day after the 2nd injection. IL-22 treatment led to a significant reduction in the stimulated salivary flow rate compared to the control group (Figure 3A), and notably increased the amount of activated, cleaved caspase-3 in the SMGs as shown by immunohistochemical staining (Figure 3B). These rapid changes induced by IL-22 were not accompanied by a significant leukocyte infiltration of SMGs based on the absence of any leukocyte focus assessed by H&E staining, with focus score being zero for both PBS- and IL-22-treated groups (Figure 3C). Thus, exogenous IL-22 can rapidly induce salivary gland tissue apoptosis and secretory dysfunction in vivo in normal C57BL/6 mice under the steady-state condition.

### 2.3. Neutralization of Endogenous IL-22 Attenuates Anti-CD3 Antibody-Induced Salivary Gland Tissue Apoptosis and Hyposalivation

**Figure 4:**
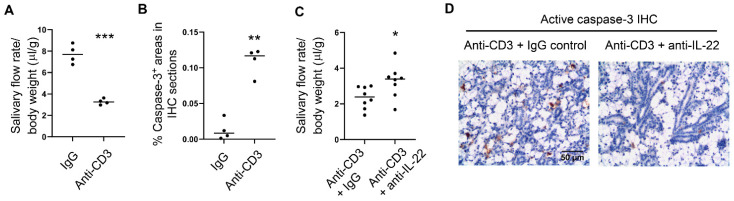
Neutralization of IL-22 attenuates salivary gland damage and dysfunction in an anti-CD3-induced acute exocrinopathy model. Anti-CD3 antibody or control IgG was i.p.-administered to 8-week-old female C57BL/6 mice on day 0 and day 2. Mice were analyzed on day 3. (**A**) stimulated salivary flow rate normalized to body weight. (**B**) immunohistochemistry of active caspase-3 in the SMGs. Magnification: 400×. (**C**) Anti-CD3 was injected in conjunction with either anti-IL-22 blocking antibody or the control IgG, and the salivary flow rate is shown. (**D**) Mice were treated as in (**C**), and the active caspase-3 levels in the SMGs are shown. In all the dot plot graphs in this figure, each black dot represents the measurement from an individual mouse. Data are the average or representative of 6 samples per group. * *p* < 0.05; ** *p* < 0.01; *** *p* < 0.001.

Systemic administration of an anti-CD3 antibody rapidly induces polyclonal T cell activation and cytokine production in mice [38,39]. We have also previously reported that such anti-CD3 treatment induces apoptosis of the SMG-and lacrimal gland tissues [32]. Here, we further demonstrated that two anti-CD3 injections in a 3-day period markedly reduced the salivary flow rate (Figure 4A), in addition to inducing tissue apoptosis (Figure 4B). To determine whether the endogenously produced IL-22 contributes to the exocrinopathy in this model, we treated female C57BL/6 mice with anti-CD3 in conjunction with a neutralizing anti-IL-22 antibody or the isotype control IgG. IL-22 neutralization significantly reduced SMG tissue apoptosis and improved salivary secretion (Figure 4C,D). Hence, endogenously produced IL-22 promotes the development of salivary gland disorder in this T cell-mediated, acute exocrinopathy model.

### 2.4. IL-22 Treatment Reduces the Viable Cell Numbers of Mouse Primary Salivary Gland Epithelial Cells Cultured In Vitro

**Figure 5:**
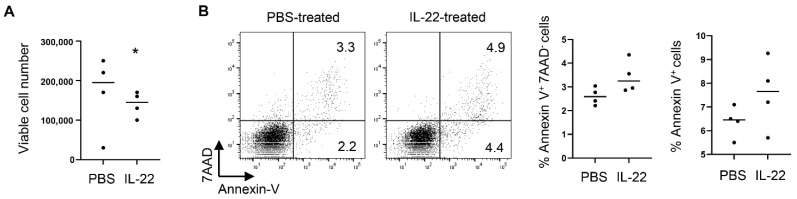
IL-22 treatment reduces the number of viable primary salivary gland epithelial cells cultured in vitro. Primary SMG epithelial cells prepared from C57BL/6 mice were cultured in the presence of PBS or recombinant IL-22 for 3 days. (**A**) Live cell numbers at the end of the culture (n = 4 per group). (**B**) Left panels, flow cytometry profile of Annexin V and 7-AAD staining patterns; right panels, percentages of Annexin V^+^7-AAD^−^ and total Annexin V^+^ cells (n = 4 per group). In all the dot plot graphs in this figure, each black dot represents the measurement from an independent cell culture group. * *p* < 0.05.

We next examined whether IL-22 can directly act on salivary gland epithelial cells. Following a published protocol [40], we established in vitro culture of primary SMG epithelial cells from the normal C57BL/6 mice. We subsequently treated these mouse SMG epithelial cells with 20 ng/mL recombinant murine IL-22 for 3 days, which markedly decreased the viable cell numbers in the culture (Figure 5A). IL-22 did not significantly affect cell apoptosis as indicated by the comparable percentage of Annexin V^+^7-AAD^−^ cells (early apoptotic cells) and total Annexin V^+^ cells (Figure 5B). Hence, IL-22 can directly act on mouse primary salivary gland epithelial cells to reduce the viable cell number in the in vitro culture.

## 3. Discussion

In this study, we provided strong evidence that IL-22, a cytokine with well-reported tissue-protective, anti-inflammatory effects on various epithelial tissues, including ocular epithelial tissues, can cause salivary gland tissue damage and dysfunction under both steady-state and SS-prone conditions. Indeed, it has been well delineated that the signaling and functional properties of IL-22 are highly complex and context-dependent [17,18,19,20]. Therefore, even though IL-22 attenuates IL-17-mediated ocular epithelial tissue inflammation and pathologies in a desiccation-induced mouse model of dry eye disease [26], we showed that it exerts a detrimental impact on salivary gland tissues in the SS disease setting, further supporting and highlighting the highly complex and disease/tissue/cell type-dependent roles of this cytokine. In addition, our study showed that the main IL-22-producing cells in salivary glands of NOD mice are the immune cells in the leukocyte foci as well as the ductal epithelial cells. This expression pattern of IL-22 is consistent with many reports showing immune cells are the main IL-22 producers in general, and also in agreement with the finding in SS patients that IL-22 is produced by both immune cells and ductal cells in the inflamed salivary glands [4]. In contrast, in the mouse model of desiccation-induced dry eye disease, the main IL-22-producing cells in the lacrimal glands are acinar cells rather than immune cells or ductal epithelial cells [26]. Therefore, our studies further strengthen the notion that both the function and production of IL-22 are disease/tissue context dependent.

Quantification of the relative protein expression levels in the immunohistochemically stained samples has certain difficulties. To enhance the rigor and reliability, the quantification was performed using the average of 6 different well-separated areas for each immunohistochemically stained sample, and the resulting values from multiple individual mouse samples were used for calculating the means and for statistical analysis. Nevertheless, it is important to note the limitations of this quantification approach.

It has been shown that the signaling and functional property of IL-22 can be influenced considerably by the surrounding cytokines/stimuli, and that type 1 IFNs and IL-17 can convert the actions of IL-22 from tissue-protective, anti-inflammatory to tissue-damaging and pro-inflammatory [22,23,24]. It is therefore unsurprising that in the SS-prone NOD mice, in which both type 1 IFNs and IL-17 are present in the target salivary gland tissues, IL-22 can exert a tissue-damaging and proinflammatory effect. However, we found that even in normal C57BL/6 mice, IL-22 can induce detectable pathological changes, including hyposalivation and caspase-3 activation, in the absence of discernable immune cell infiltration of salivary glands. These results suggest that IL-22 effects on salivary gland tissues may be detrimental in nature even under steady-state, non-inflammatory conditions. We have previously shown that IL-6, a cytokine with prominent pro-inflammatory activities in many inflammatory and autoimmune conditions, plays a tissue-protective and anti-inflammatory role in an SS disease model and the anti-CD3-induced acute exocrinopathy model [32]. It is hence interesting that both IL-6 and IL-22 adopt their less commonly reported activities in the salivary gland tissues. The signaling events elicited by IL-6 and IL-22 have many similarities, particularly the activation of STAT3 signaling pathway, but the distinct effects of these two cytokines in salivary gland tissues prompt future investigations into the underlying mechanisms and the unique features of salivary gland tissues.

It was previously shown that IL-22 can upregulate IL-18 production from intestinal epithelial tissues to enhance immune cell IFNγ production in *T. gondii*-induced ileitis [21]. Here we found that it has a similar impact on salivary gland tissues, upregulating the expression of IFNγ and the IFNγ/Th1-promoting IL-18. Hence, although IL-22 can induce salivary gland damage and dysfunction to some degree even under steady-state conditions, the upregulation of IL-18 and IFNγ in the SS-prone condition by IL-22 likely further exacerbates and sustains these tissue damages and dysfunctions, which is a hypothesis to be investigated. In addition, the signaling events and molecular mechanisms by which IL-22 upregulates IL-18 and IFNγ expression in salivary gland epithelial tissues require further elucidation.

A notable finding in this study is the identification of IL-22-producing B cells in salivary glands and an increase in these cells in SS-stricken NOD mice compared to the Balb/c mice. It has been shown that TNFα, IL-23 and IL-6 are the major cytokines promoting IL-22 production from T cells and innate lymphoid cells [17,18,19,20], and all these cytokines are produced at an elevated amount in SS inflammatory conditions [6,9,10,15,32,41,42]. It is therefore conceivable that some of these cytokines contribute to the excessive IL-22 production by B cells and other cell types in salivary glands in the SS setting, which will require experimental validation in the future. It was previously reported that a significant portion of mononuclear cells in salivary glands of SS patients, and those in the non-Hodgkin’s lymphoma (NHL) lesions of SS patients, can produce IL-22 [4]; it will be interesting to examine if some of these IL-22-producing cells in SS patients are B cells. More importantly, the functional significance of endogenous IL-22, including B cell-derived IL-22, in the pathogenesis and persistence of SS disease should be determined using in vivo loss-of-function approaches with cell type-specific IL-22 ablation. In a viral infection-induced mouse model of SS, IL-22 was shown to be essential for the optimal B cell accumulation and TLO formation in the salivary glands and for the consequent autoantibody production, with the effects associated with upregulation of CXCL12 and-13 production by tissue cells in the TLOs [27]. In SS patients with NHL, IL-22R1 is expressed by some of the B cells in the NHL lesions, suggesting the possibility that these B cells can directly respond to IL-22 [4]. By both producing IL-22 and responding to IL-22, B cells may perpetuate their own accumulation in the TLOs to reinforce the production of autoantibodies and the progression of NHL.

In this study, we demonstrate the importance of endogenously produced IL-22 in a mouse model of short-term, self-resolving inflammation induced by systemic administration of anti-CD3 antibody. Studies by our group and others have shown that i.p.-administration of anti-CD3 rapidly induces polyclonal T cell activation and cytokine production in mice, leading to inflammation of multiple organs and tissues, including intestines, lungs and liver [38,39,43,44]. The self-limiting feature of the inflammation in this model is in part due to the trans-differentiation of intestinal Th17 cells into regulatory T cells [44]. We previously showed that this model can also serve as an acute exocrinopathy model because of the readily detectable apoptosis of SMG- and lacrimal gland tissues apart from inflammation of other organs [32]. We postulate that the salivary gland-affecting IL-22 in this model could be derived from local IL-22-producing immune cells and/or those generated at other sites, such as intestines, that migrated into salivary glands. Moreover, the systemic elevation of IL-22 in the blood and the lymphatic system may also exert an impact on salivary gland tissues.

Our finding that IL-22 treatment reduced the number of live mouse salivary gland epithelial cells cultured in vitro without significantly affecting cell death suggests that the pro-apoptotic effect of IL-22 on salivary gland tissues observed in vivo likely requires the presence of additional factors. Whether IL-22-induced reduction in the viable salivary gland epithelial cells in these cultures resulted from the impairment of cell proliferation will require further exploration. Interestingly, a recent study demonstrates that IL-22 promotes chronic colitis through facilitating the endoplasmic reticulum stress response in colonic epithelial cells [25]. Hence, the additional impacts of IL-22 on salivary tissue homeostasis, integrity and function and the full range of the underlying mechanisms, such as affecting the endoplasmic reticulum stress response, all await future in-depth investigations.

## 4. Materials and Methods

### 4.1. Mice

C57BL/6 mice, Balb/c mice and non-obese diabetic (NOD) mice (NOD/ShiLtJ strain) were purchased from the Jackson Laboratory (Bar Harbor, ME, USA) and were housed in the specific pathogen-free animal facility at the Forsyth Institute. All the experimental protocols were approved by the Institutional Animal Care and Use Committee of the Forsyth Institute. All the procedures were performed in compliance with the National Institute of Health guidelines for the care and use of laboratory animals.

### 4.2. Cytokine and Antibodies

Recombinant murine IL-22 was purchased from Peprotech (Cranbury, NJ, USA). Purified anti-mouse CD3ε (145-2C11) and its isotype control Armenian hamster IgG for the injection were obtained from BioLegend (San Diego, CA, USA) and BioXCell (Lebanon, NH, USA), respectively. Purified monoclonal anti-mouse IL-22 (IL22JOP) and its isotype control rat IgG2a used for in vivo administration were purchased from eBioscience and BioXCell, respectively. For immunohistochemistry, rabbit polyclonal anti-claudin-1 antibody was purchased from AbCam (Waltham, MA, USA). For flow cytometry, fluorescence conjugated antibodies specific for CD45 (clone 30-F11), CD4 (GK1.5), CD8α (536-7), CD19 (1D3), TCR-β (H57-597), IL-22 (Poly5164) and CD16/32 (clone 93) antibodies were purchased from BioLegend.

### 4.3. In Vivo Administration of Recombinant IL-22 and Anti-IL-22 Antibody

To assess the effects of exogenous IL-22 on submandibular glands (SMGs) in vivo, recombinant murine IL-22 (1 µg, PeproTech) was directly injected into SMG of 7–12 weeks old female C57BL/6 mice and 8–9 weeks old female NOD mice every other day for 3 days. To determine the effects of endogenous IL-22, Anti-CD3 (145-2C11, 20 μg) and anti-IL-22 (IL22JOP, 100 μg) antibodies were intraperitoneally injected into 8-weeks-old female C57BL/6 mice every other day for 3 days. The analyses were performed 3 or 24 h after the last injection.

### 4.4. Measurement of Salivary Flow Rate

Non-anesthetized mice were weighed and intraperitoneally injected with 100 μL PBS-based secretagogue solution containing isoproterenol (1 mg/mL) and pilocarpine (2 mg/mL). One min after secretagogue injection, saliva was collected continuously for 5 min from the oral cavity of mice with a micropipette. The volume of saliva was measured and normalized to the body weight.

### 4.5. Histology and Immunohistochemistry

Harvested SMG tissues were fixed in 4% paraformaldehyde, embedded in paraffin, and sectioned to 5 μm thickness. The sections were subsequently stained with hematoxylin and eosin (H&E) for the detection of leukocyte infiltration. For immunohistochemistry staining, the sections were de-paraffinized and stained with rabbit anti-claudin-1 polyclonal antibody (AbCam) at 4 °C overnight using VECTASTAIN Elite ABC Kit (Vector Laboratories, Newark, CA, USA), or with the Active caspase-3 using SignalStain^®^ Apoptosis (Cleaved Caspase-3) IHC Detection Kit (Cell Signaling Technology, Danvers, MA, USA), following the manufacturer’s instructions. Images were recorded at 400× magnification under a light microscope. The positively stained areas in the stained sections were quantified using the Image J 1.45 s software. Briefly, 6 images per sample were saved as RGB Tiff files and the brown-stained areas were segmented using appropriate color thresholding. The percentage of thresholded areas in each image was measured and used for further calculation and statistical analysis.

### 4.6. Flow Cytometry

Single cells from SMGs or submandibular lymph nodes (smLNs) were prepared and incubated with anti-CD16/32 antibody to block non-specific binding. The cells were then stained with a combination of fluorescence-conjugated antibodies to surface markers CD4 (GK1.5), CD8α (536-7), CD19 (1D3) at 4 °C for 30 min. The cells were then fixed, permeabilized, incubated with anti-IL-22 antibody for 30 min, washed and analyzed using a FACS Arial III flow cytometer (BD, Franklin Lakes, NJ, USA). Data were further processed with the FlowJo V10 software (FlowJo, Ashland, OR, USA).

### 4.7. Real-Time RT-PCR

Total RNA from SMG tissues was isolated using the RNeasy Micro kit (Qiagen, Germantown, MD, USA) and transcribed into complementary DNA (cDNA) with M-MLV reverse transcriptase (Promega, Madison, WI, USA). Real-time PCR was performed using SYBR Green master mix (Qiagen) for 40 cycles with annealing and extension temperature at 60 °C, on a Roche LightCycler 480 Real-Time PCR system. Primer sequences are: mouse β-actin forward, 5′-TGGATGACGATATCGCTGCG-3′, reverse, 5′-AGGGTCAGGATACCTCTCTT-3′; AQP5 forward, 5′-ATTGCT GGAGCAGGCATCCT-3′, reverse, 5′-ATTAACTCCACCACCACGGC-3′; Occludin forward, 5′-AGTCCACCTCCTTACAGACC-3′, reverse, 5′- AAGAGTACGCTGGCTGAGAG-3′; Claudin-1 forward, 5′-CAAAGCACCGGGCAGATACA-3′, reverse, 5′-CCAGCAGGATGCCAATTACC-3′; Mcl-1 forward, 5′-TTGTAAGGACGAAACGGGACT-3′, reverse, 5′-ACATTTCTGATGCCGCCTTCT-3′; Bcl-xL forward, 5′-CACCTAGAGCCTTGGATCCA-3′, reverse, 5′-TTGAAGCGCTCCTGGCCTTT-3′; Bax forward, 5′-GAGACACCTGAGCTGACCTT-3′, reverse, 5′-GTTCATCTCCAATTCGCCGG-3′; IFN-γ forward, 5′-GGATGCATTCATGAGTATTGC-3′, reverse, 5′-CCTTTTCCGCTTCCTGAGG-3′; IL-18 forward, 5′-GACTCTTGCGTCAACTTCAAGG-3′, reverse, 5′-CAGGCTGTCTTTTGTCAACGA-3′. Sequences for other primers will be provided upon request. The gene expression level was normalized to that of β-actin.

### 4.8. In Vitro Culture and Treatment of Primary Mouse SMG Cells

We isolated and cultured the primary mouse SMG cells. Briefly, harvested SMG tissues from 8 weeks old female C57/BL6 mice were digested with 5 mL DMEM containing 10% FBS, 1% penicillin-streptomycin and collagenase (8 mg/mL). Then cells were filtered through a 100 μm nylon mesh and digested with 0.05% Trypsin-0.02% EDTA. The digested cells were re-suspended with epidermal keratinocyte medium, CnT-07 (CELLnTEC, Bern, Switzerland). Primary mSMG cells were used between passages 5 to 11. The primary mSMG cells were seeded at a density of 2 × 10^5^ cells per well in 24-well culture plates in the presence or absence of recombinant murine IL-22 (20 ng/mL) for 3 days.

### 4.9. Annexin V and 7-AAD Staining for Apoptosis Detection

Viable cell numbers were counted based the Trypan blue exclusion (Corning Inc., Corning, NY, USA). Cell apoptosis was detected by staining with Annexin V-Fluorescein isothiocyanate (FITC) and 7-AAD (BioLegend). Briefly, 2 × 10^5^ primary mSMG cells were incubated with recombinant murine IL-22 (20 ng/mL) for 3 days. The cells were subsequently stained with Trypan blue solution for viable cell enumeration, or incubated with Annexin V-FITC plus 7-AAD (BioLegend) for apoptosis detection. The cells were then analyzed using a BD FACSAria III cell sorter/flow cytometer and the Flowjo software to determine the percentage of cells stained positive for Annexin V and/or 7-AAD.

### 4.10. Statistical Analysis

Statistical significance was determined by One-way ANOVA or two-tailed Student’s *t*-test as appropriate. *p* values smaller than 0.05 were considered as statistically significant.

## 5. Conclusions

This study provides strong evidence for a detrimental impact of IL-22 on the integrity and function of salivary gland tissues. It also defines several critical cellular effects of IL-22 and identified B cells as important sources of IL-22 in the SS disease setting. This study hence yields new insights into SS pathogenesis and IL-22 function and regulation, which could be a potential new therapeutic target for salivary gland exocrinopathy caused by SS and possibly other disease conditions.
